# Potential Therapeutic Target Protein Tyrosine Phosphatase-1B for Modulation of Insulin Resistance with Polyphenols and Its Quantitative Structure–Activity Relationship

**DOI:** 10.3390/molecules27072212

**Published:** 2022-03-29

**Authors:** Prangya Rath, Anuj Ranjan, Arabinda Ghosh, Abhishek Chauhan, Manisha Gurnani, Hardeep Singh Tuli, Hamza Habeeballah, Mustfa F. Alkhanani, Shafiul Haque, Kuldeep Dhama, Naval Kumar Verma, Tanu Jindal

**Affiliations:** 1 Amity Institute of Environmental Sciences, Amity University, Noida 201303, India; prangyarath71@gmail.com (P.R.); manishagurnani10@gmail.com (M.G.); 2 Academy of Biology and Biotechnology, Southern Federal University, 344006 Rostov-on-Don, Russia; 3 Microbiology Division, Department of Botany, Gauhati University, Guwahati 781014, India; 4 Amity Institute of Environmental Toxicology Safety and Management, Amity University, Noida 201303, India; akchauhan@amity.edu (A.C.); tjindal@amity.edu (T.J.); 5 Department of Biotechnology, Maharishi Markandeshwar (Deemed to be University), Mullana, Ambala 133207, India; hardeep.biotech@gmail.com; 6 Faculty of Applied Medical Sciences, King Abdulaziz University, Rabigh Branch, Rabigh 25732, Saudi Arabia; hhabeeballah@kau.edu.sa; 7 Emergency Service Department, College of Applied Sciences, AlMaarefa University, Riyadh 11597, Saudi Arabia; mkhanani@mcst.edu.sa; 8 Research and Scientific Studies Unit, College of Nursing and Allied Health Sciences, Jazan University, Jazan 45142, Saudi Arabia; shafiul.haque@hotmail.com; 9 Faculty of Medicine, Bursa Uludağ University Görükle Campus, Nilüfer 16059, Turkey; 10 Division of Pathology, ICAR-Indian Veterinary Research Institute, Izatnagar, Bareilly 243122, India; kdhama@rediffmail.com; 11 Homeopathy, Ministry of Ayush, Ayush Bhawan, B Block, GPO Complex INA, New Delhi 110023, India; drnaval.kumar@gmail.com

**Keywords:** catalytic active site, diabetes, docking, insulin resistance, molecular dynamic simulation, polyphenols, QSAR

## Abstract

The increase in the number of cases of type 2 diabetes mellitus (T2DM) and the complications associated with the side effects of chemical/synthetic drugs have raised concerns about the safety of the drugs. Hence, there is an urgent need to explore and identify natural bioactive compounds as alternative drugs. Protein tyrosine phosphatase 1B (PTP1B) functions as a negative regulator and is therefore considered as one of the key protein targets modulating insulin signaling and insulin resistance. This article deals with the screening of a database of polyphenols against PTP1B activity for the identification of a potential inhibitor. The research plan had two clear objectives. Under first objective, we conducted a quantitative structure–activity relationship analysis of flavonoids with PTP1B that revealed the strongest correlation (R^2^ = 93.25%) between the number of aromatic bonds (naro) and inhibitory concentrations (IC_50_) of PTP1B. The second objective emphasized the binding potential of the selected polyphenols against the activity of PTP1B using molecular docking, molecular dynamic (MD) simulation and free energy estimation. Among all the polyphenols, silydianin, a flavonolignan, was identified as a lead compound that possesses drug-likeness properties, has a higher negative binding energy of −7.235 kcal/mol and a pKd value of 5.2. The free energy-based binding affinity (ΔG) was estimated to be −7.02 kcal/mol. MD simulation revealed the stability of interacting residues (Gly183, Arg221, Thr263 and Asp265). The results demonstrated that the identified polyphenol, silydianin, could act as a promising natural PTP1B inhibitor that can modulate the insulin resistance.

## 1. Introduction

Insulin signaling is a complex integrated network which initiates with the binding of the insulin molecule to the insulin receptors on the cellular surface. Insulin receptors (IR) belong to the protein tyrosine kinase (PTKs) group of the family that constitutes two extracellular α-subunits and two transmembranic β-subunits joined to each other by disulfide bonds [1]. The binding of the insulin molecule to the α-subunit of the IR leads to conformational changes in the β-subunits, thereby leading to its autophosphorylation at tyrosine residues [2]. This triggers further downstream insulin receptor substrates (IRS) to carry forward the signaling into the cell, which is followed by the recruitment and activation of phosphatidylinositol 3-kinase (PI3K) and its downstream protein kinase B (PKB, also known as Akt) pathway. The latter is then responsible for carrying out many metabolic actions related to insulin signaling, such as the increase in the expression and translocation of the glucose transport channel 4 (GLUT-4), the increase in the stimulation of the glycogen synthesis pathway and the inhibition of gluconeogenesis [3,4]. At cellular levels, the activity of the IR is tightly regulated, as unchecked activation or inactivity would lead to profound metabolic consequences. The normal mechanism of insulin signaling and leptin action in cells is blocked/hindered by a phosphatase, namely the protein tyrosine phosphatase 1B (PTP1B) protein. PTP1B acts either by dephosphorylating the tyrosine residues of the IR, rendering it inactive, or by dephosphorylating the IRS-1/2 and inhibiting their interactions with downstream signaling molecules [1,3]. 

PTP1B is a widely expressed cytosolic phosphatase protein whose structure has been well studied. It has a molecular weight of nearly 50 kDa which is encoded by the PTPN1 gene. The three-dimensional structure of the protein consists of 435 amino acid residues having eight α-helices and eleven β-strands and is organized into three regions. Amino acid residues 1–300 form the N-terminal catalytic region or the PTP domain, amino acid residues 300–400 form the regulatory region, while residues 400–435 form the C-terminal membrane localization region [5]. The R-loop (Val113–Ser118), lysine loop (Leu119–Cys121), WPD loop (Thr177–Pro185), S-loop (Ser201–Gly209), Q-loop (Ile261–Gln262), α3 helix (Glu186–Glu200), α6 helix (Ala264–Ile281) and α7 helix (Val287–Ser295) play critical roles in the dephosphorylation of phosphotyrosine. The catalytic active site of the protein is made up of the amino acid residues His214–Arg221 (His214-Cys215-Ser216-Ala217-Gly218-Ile219-Gly220-Arg221), containing the active site nucleophile Cys215 residue and Arg221, which bind with the substrate and stabilizes the transitive state. Other residues, which form the sides of the catalytic cleft and contribute to the catalysis and substrate recognition, are Tyr46, Val49, Lys120, Asp181, Phe182 and Gln262 [3,6]. The WPD loop and S-loop play a crucial role in the mechanism of the conformation change of the protein, which is essential for the activity of the enzyme, while the R-loop assists in the substrate binding [7,8]. The protein dephosphorylates the phosphorylated tyrosine residues on the insulin receptors, thereby hindering the further downstream signaling pathway. Previous studies have shown that a deficiency of PTP1B in mice models has led to enhanced insulin sensitivity by increasing the phosphorylation of the insulin receptor in tissue, as well as an increased basal metabolic rate, a low adiposity and a total energy expenditure [9,10]. The design of the PTP1B inhibitors has been challenging. A number of drug design studies have focused on inhibitors targeting the active site of PTP1B, as it is a conserved region [11,12,13,14]. The binding of a compound either in an active site or to a secondary noncatalytic region adjacent to the active site is useful to achieve the selectivity for PTP1B [15].

Studies have confirmed that the overexpression of PTP1B in cells has led to the reduction in glucose uptake, the translocation of GLUT-4 channels, the decrease in IR and IRS-1 tyrosine phosphorylation, in PI3K levels and in glycogen synthesis in adipose tissue cells, and that this regulation of insulin signaling has been observed to be tissue-specific. Similarly, work done on L6-myocytes and FaO hepatoma cell lines have shown that the overexpression of PTP1B has blocked the insulin-stimulated tyrosine phosphorylation of both IR as well as IRS-1, and has also affected downstream proteins like PI3K and Akt [16,17,18]. Inhibiting PTP1B improved the insulin sensitivity in obese and insulin-resistant ob/ob mice, reduced the hyperinsulinemia and normalized blood glucose levels in the liver and skeletal muscle cells of obese db/db mice. It also downregulated key liver gluconeogenic enzymes [19]. Its inhibition has been observed to facilitate wound healing in diabetic patients by promoting angiogenesis and tissue regeneration while decreasing inflammation and microbial infection at the wound site [20]. It has been studied that specifically targeting the PTP1B protein in the liver appears to treat metabolic syndromes by not only improving whole-body insulin sensitivity, but also decreasing lipid deposition in the liver [21]. The phosphatase property of the protein makes it one of the key negative regulators of the insulin signaling pathway and has been a causal factor in the emergence of T2DM. It has therefore been a key target protein for developing therapeutic implications. Therefore, PTP1B inhibition has gained the attention of the pharmaceutical industry. Synthetic candidate drugs, including the thiazolidinedione class of drugs and its derivatives, ertiprotafib, ISIS 113715, ISIS-PTP1BRx, trodusquemine, benzofuran, benzothiophene biphenyls, vanadium complexes, aminobenzoic acid, phosphonic acid, carboxylic acids, sulfonic acids, phosphonodifluoromethyl, phenylalanine derivatives, imides, etc., have been studied for ameliorating insulin resistance by inhibiting PTP1B [22,23,24,25]. However, the associated side effects and safety profiles of synthetic drugs have been an important question. As a result, there is a rising importance in the usage of natural inhibitors of PTP1B with minimal/no side effects. Natural inhibitors are composed of terpenes, phenolic compounds, flavonoids, bromophenols, alkaloids and other groups [26,27]. The asperentin analog produced by a fungi strain from a deep-sea environment, namely Aspergillus sydowii, has been studied to inhibit PTP1B at an IC_50_ of 2 μM [28]. Trivaric acid, produced by microbes, showed a strong PTP1B-inhibitory activity with an IC_50_ of 173 nM, enhanced insulin sensitivity, improved stimulation of the IR/IRS/Akt/GLUT-2 pathway and improved glucose uptake in liver HepG2 cell lines [29]. Phytochemicals like oleanolic acid, ursolic acid, corosolic acid, 23-hydroxyursolic acid and rhododendric acid A possess a high inhibitory activity against PTP1B, with an IC_50_ = 9.5 ± 0.5 μM, 3.1 ± 0.3 μM, 7.0 ± 0.6 μM, 7.4 ± 0.6 μM and 6.3 ± 0.6 μM, respectively [30,31,32]. Extracts from the seeds of *Silybum marianum* are rich in flavonolignans that have proved to be a novel class of PTP1B inhibitors and have the potential for antidiabetic drug development [33].

This study aims at identifying natural polyphenol(s), an important class of phytochemicals which could be potential inhibitors of the PTP1B protein. In this paper, we have targeted the catalytic active site of the protein for its binding with polyphenols, analyzed the ligand binding efficiencies and the binding affinity (ΔG) energies. The polyphenols were screened for their drug-likeness properties by dock score/binding energies. The top ranked polyphenols were studied for their respective interactions with the key residues in the catalytic pocket of PTP1B. The Schrödinger tool was used for studying the binding free energy (MMGBSA) and the ligand-binding efficiency was calculated using KDeep. After screening, a list of polyphenols was identified as potential inhibitors of PTP1B. The best docked pose of the top hit ligand–protein complex was subjected to molecular dynamic (MD) simulation and was analyzed in detail. The study confirms that selective and permeable inhibitors can be identified by targeting the active site of PTP1B. It was observed that many flavonoids bound to the PTP1B active site. Therefore, the paper also emphasizes the quantitative structure–activity relationship (QSAR) models, which were generated to analyze the correlation between the structure and activity of flavonoids with PTP1B. The structure–activity relationship models generated can be used for predicting the pharmacophore parameters.

## 2. Results

### 2.1. QSAR Model Analysis

**Figure 1:**
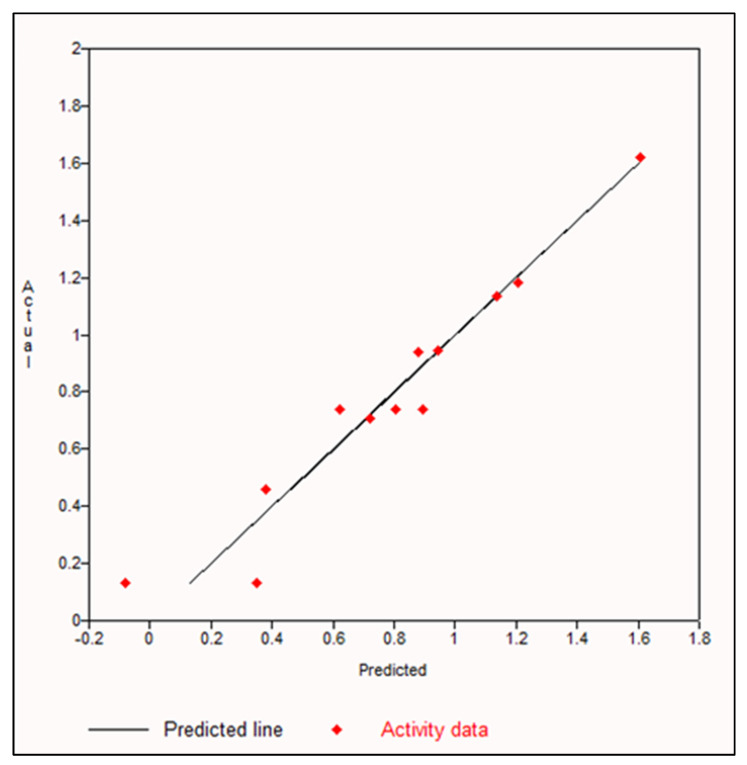
Graph depicting the correlation analysis between increase in number of aromatic bonds and PTP1B inhibition.

The QSAR models were developed by employing multiple linear regression to calculate the predicted LogIC_50_ value for the flavonoid molecules and the PTP1B protein. The differences in the experimental and predicted data were observed to be small. Among all the parameters used for this study, increasing the number of aromatic bonds (naro) was observed to show the maximum correlation with the PTP1B activity in the 25:75 ratio set, at 93.25% in training set data and 91.12% in test set data. Figure 1 shows the correlation graph between the number of aromatic bonds and the PTP1B inhibition activity. Table 1 shows the R^2^ and adjusted R^2^ of the training set and test set models generated. The value of R^2^–adjusted R^2^ was observed to be least in the 25:75 ratio set. High R^2^ values and less difference in the R^2^–adjusted R^2^ indicates a better QSAR equation [34]. Equation (1) shows the model equation generated.

LogIC_50_ = 1.144 + 0.0490 × (MW) + −6.481 × (HBA) + −4.669 × (HBD) + −0.2106      × (nrot) + 0.1516 × (naro) + 0.442 × (TPSA) + −1.807 × (LogP)(1)

### 2.2. Lipinski’s Rule of Five

A total of 173 bioactive compounds/phytochemicals subjected to Lipinski’s rule of five by using the DruLiTo software resulted in the selection of 119 phytochemicals which met the primary criteria of drug-likeness properties. It is a key consideration while selecting compounds during the early stages of drug discovery, as it is useful in shortlisting the compounds with favorable molecular properties. Lipinski’s rule of five sets ‘drug likeness’ guidelines for new molecular entities (NMEs) [35]. According to this rule, a compound can be considered as a drug candidate if it fulfils the following criteria: (i) a molecule with a molecular mass less than 500 Daltons; (ii) not more than five hydrogen bond (H-bond) donors (HBD); (iii) not more than ten H-bond acceptors (HBA); (iv) a molar refractivity ranging from 40 to 130; (v) an octanol–water partition coefficient or high lipophilicity, denoted as Log *P*, should not be greater than five [36,37]. 

### 2.3. Docking Results

**Figure 2:**
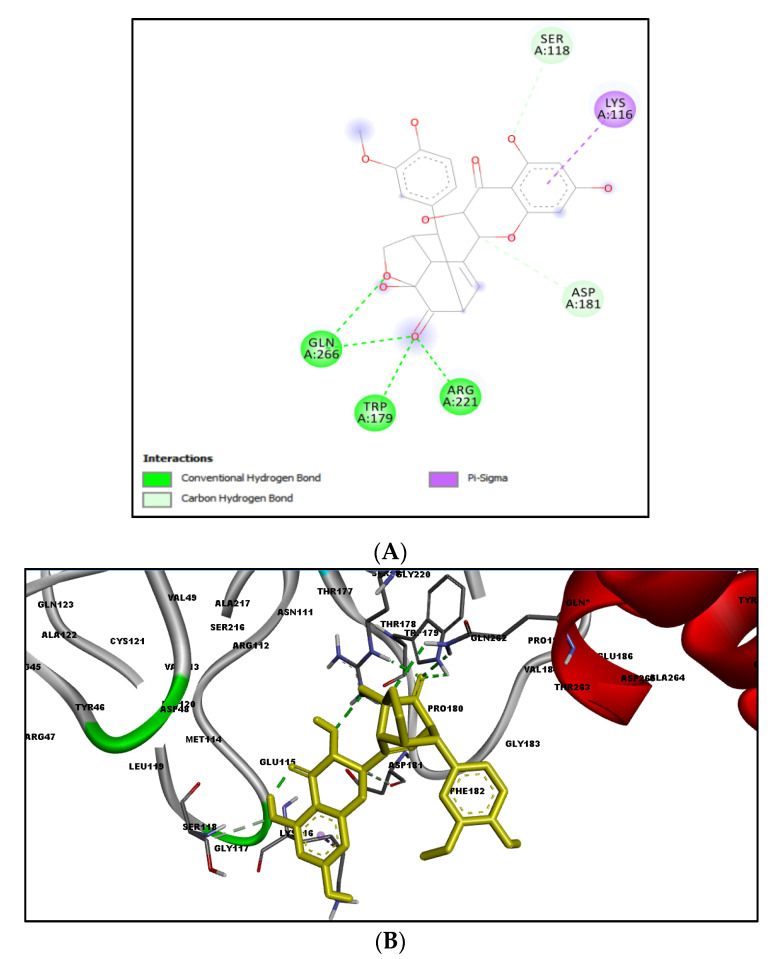
Molecular interactions of silydianin with the catalytic active site of PTP1B protein in Schrödinger’s suite. (**A**) Two-dimensional view. (**B**) Three-dimensional view. The dashed lines represent the bond formations between the ligand and amino acids (protein). Trp179, Arg221 and Gln266 form conventional H-bonds (dark green color), Ser118 and Asp181 form carbon–hydrogen bonds (light green color), while Lys116 forms Pi-sigma bonds (purple color).

**Figure 3:**
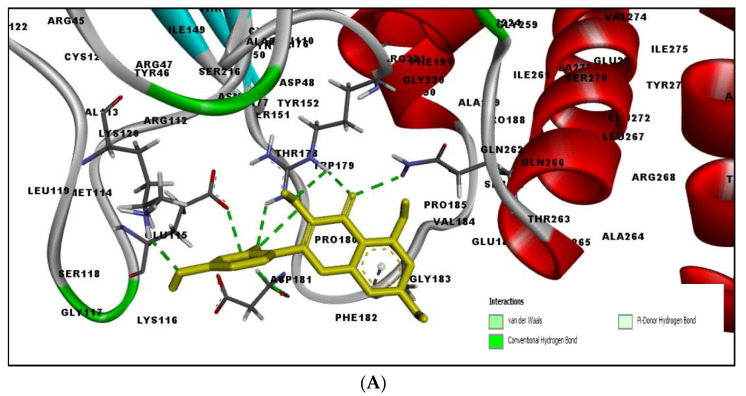

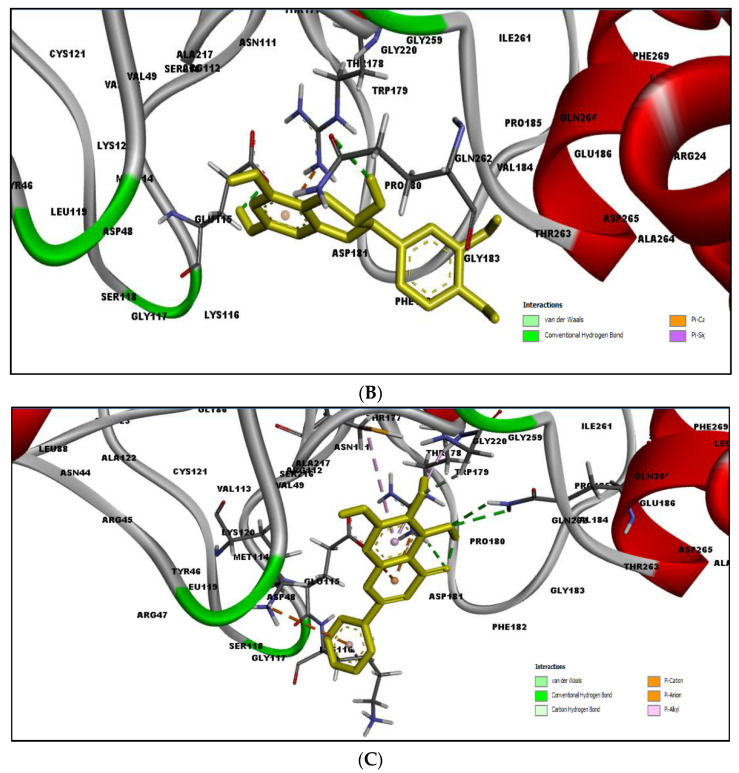
Three-dimensional view depicting molecular interaction of few top shortlisted polyphenols with the catalytic active site of PTP1B protein. (**A**) Morin (Glu115, Lys120, Asp181, Gly183, Arg221, Gln266). (**B**) Cianidanol (Glu115, Arg221, Gln262, Thr263). (**C**) Baicalein (Glu115, Lys116, Lys120, Cys215, Gly220, Arg221, Gln266). The dashed lines represent the bond formations between ligand and amino acids (protein). Color code representation: dark green—conventional H-bond; light green—van der Waals; pale green—Pi-Donor H-bond; purple—Pi-sigma bonds; dark orange—Pi-anion; light orange—Pi-cation; pink—Pi-alkyl.

Table 2 shows the result of the docking by Schrödinger’s suite (docking based virtual screening and extra precision docking), which showed the docking score, XPGScore and MMGBSA energy. The result arranged the phytochemicals according to their increasing binding energies. The table was sorted to analyze the polyphenols. The top hit polyphenol was silydianin. It bound to the active site of the PTP1B protein with a −7.235 kcal/mol docking score in Schrödinger’s suite. The molecular interactions of silydianin with the catalytic active site of the PTP1B protein in two-dimensions and three-dimensions have been represented in Figure 2. The two-dimensional structural analysis revealed that Trp179, Arg221 and Gln266 formed H-bonds with the hydroxyl and ketone groups, while Ser118 and Asp181 formed carbon–hydrogen bonds and Lys116 formed a Pi-sigma bond with the PTP1B active site cavity. The top-ranked polyphenols showing a good docking score in the virtual screening workflow and the extra precision docking of Schrödinger’s suite were shortlisted. Figure 3 represents the molecular interaction of a few top shortlisted polyphenols with the catalytic active site of the PTP1B protein. 

**Figure 4:**
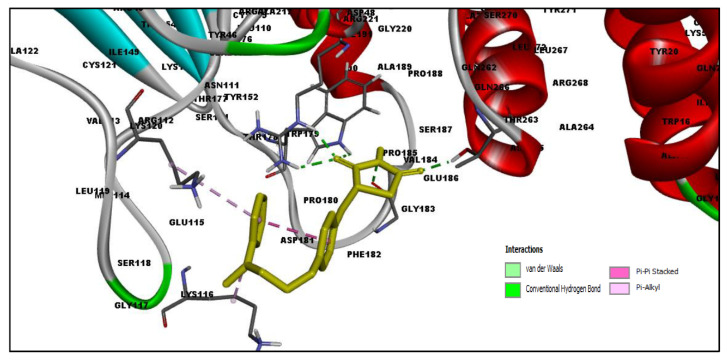
Three-dimensional binding pattern of synthetic drug rosiglitazone with the catalytic active site of PTP1B protein. The dashed lines represent the bond formations between ligand and amino acids (protein). Trp179, Gly183, Arg221 and Thr263 form conventional H-bonds (dark green color), Lys116 and Lys120 form Pi-alkyl (pink color).

Common synthetic drugs included for the study are acetohexamide, chloropropamide, glibenclamide, glipizide, metformin, rosiglitazone, tolazamide, tolbutamide and topiramate. Rosiglitazone was the only synthetic drug qualifying for the Schrödinger docking, with a docking score of −5.432 kcal/mol. Figure 4 shows the binding pattern of the rosiglitazone drug with the catalytic active site of the PTP1B protein.

### 2.4. MD Simulation Results

**Figure 5:**
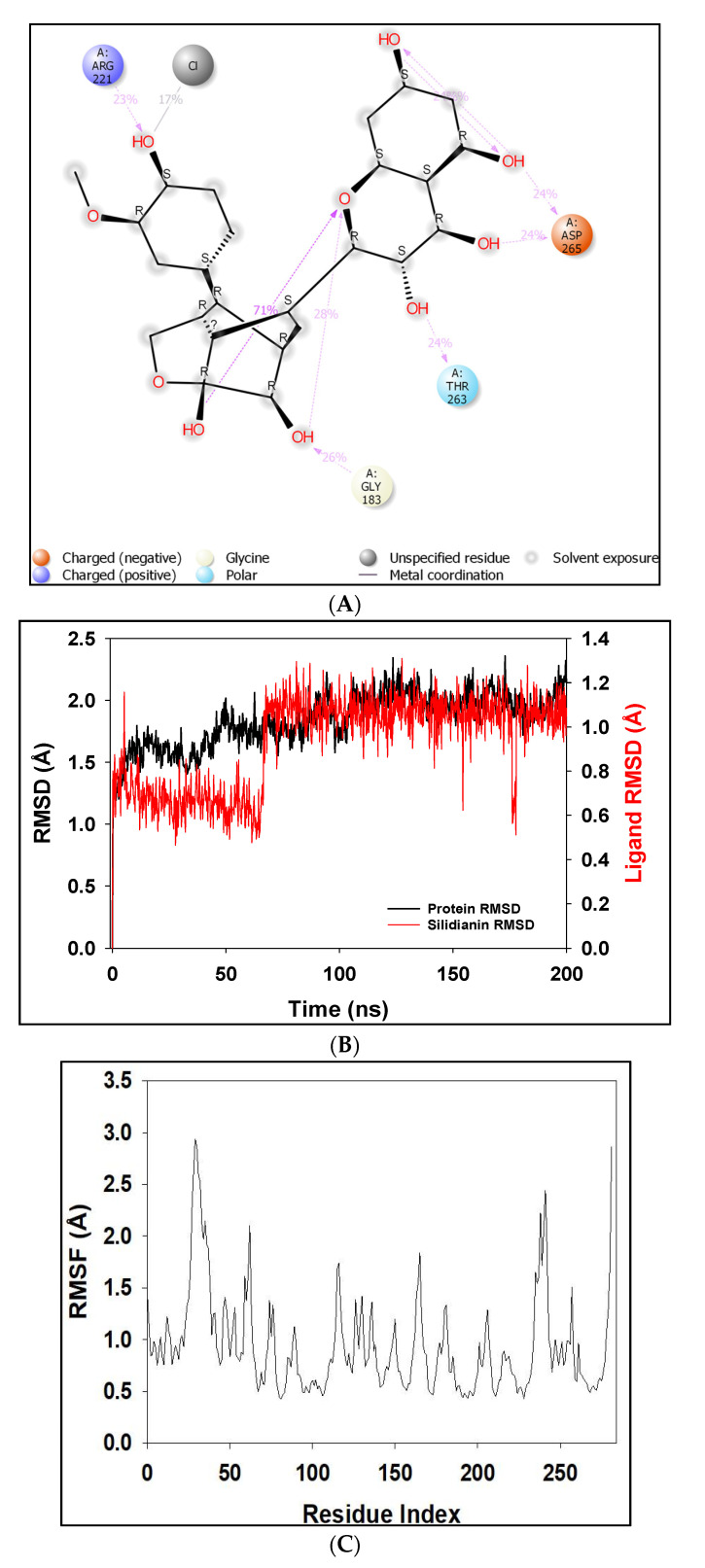

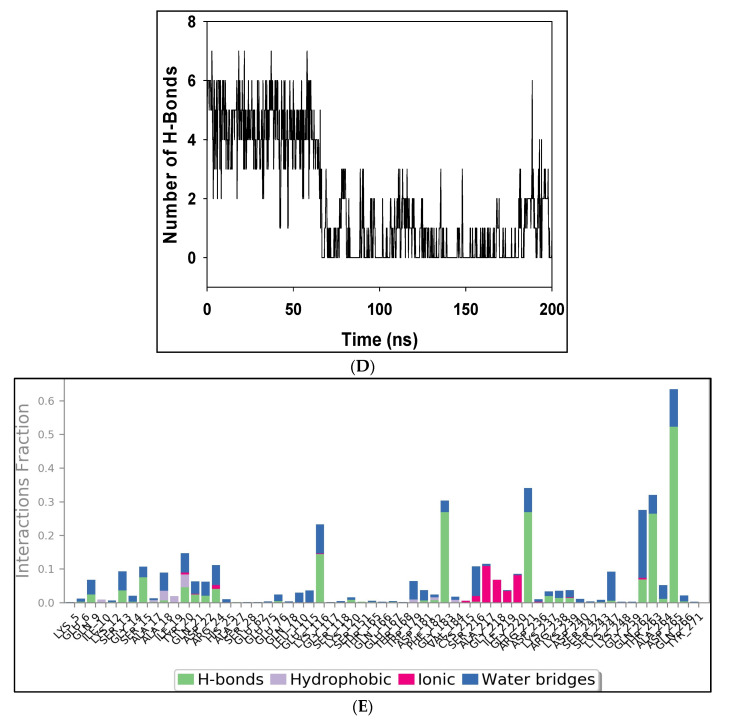
The detailed interaction of PTP1B–silydianin along with the water molecule. (**A**) Key residues showing >5.0% of the simulation time in the selected trajectory. (**B**) Protein–ligand RMSD lot for 200 nsec depicting complex stability from ~70 nsec to ~200 nsec. (**C**) RMSF graph of the complex highlights that the complex did not distort during the process. (**D**) Number of hydrogen bonds formed throughout the simulation between protein and ligand (**E**) Histogram chart of protein–ligand contact of the four types of bonds.

**Figure 6:**
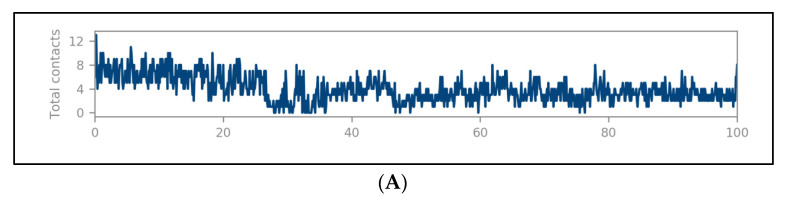

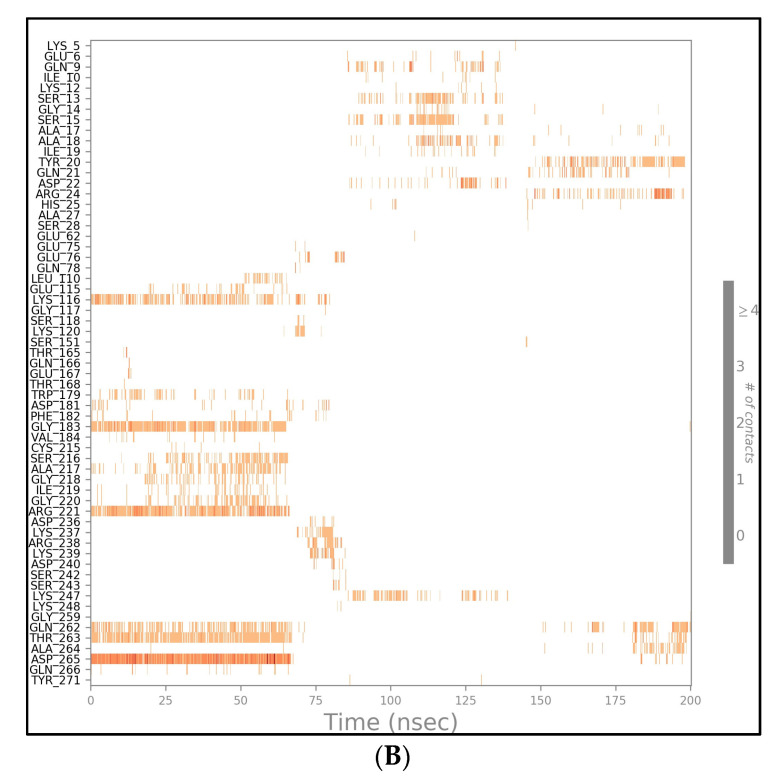
A 200 nsec timeline representation of the protein–ligand (PTP1B–silydianin) contacts. (**A**) Total number of specific contacts the PTP1B protein makes with the silydianin over the course of the trajectory. (**B**) PTP1B residues which interact with the silydianin in each trajectory frame.

**Figure 7:**
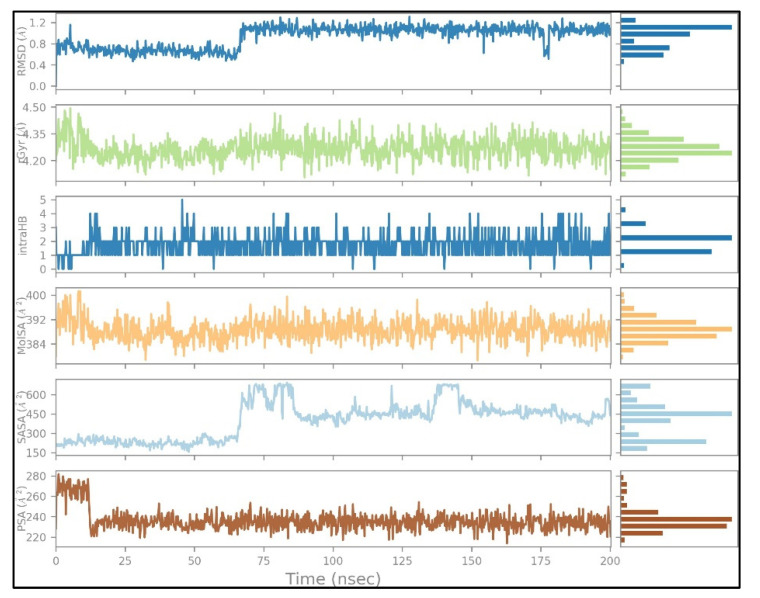
Ligand (silydianin) properties during the 200 nsec simulation trajectory.

**Figure 8:**
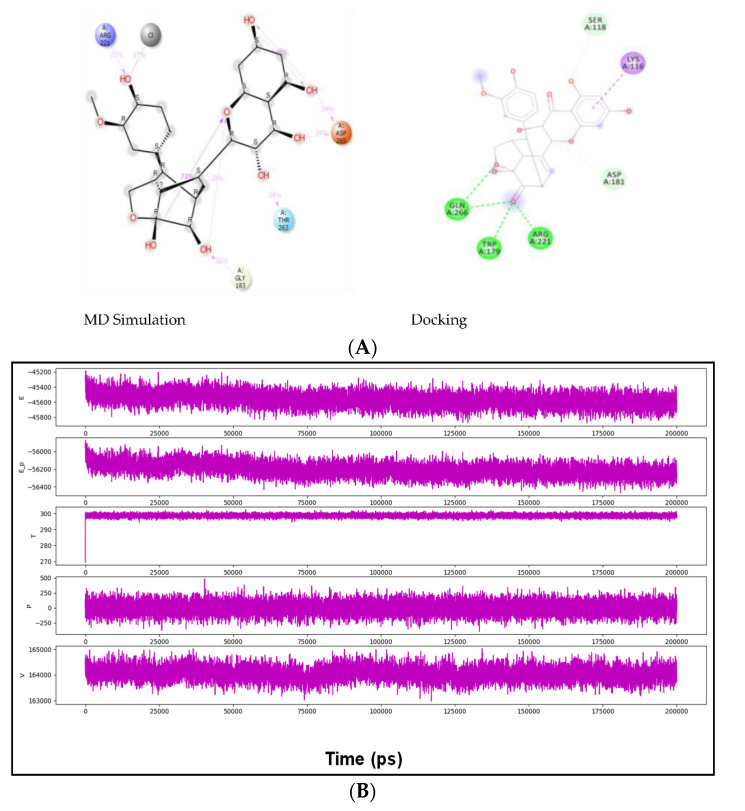
(**A**) A comparison of the interaction of the docking result and the simulation result showing that Arg221 was observed in both. (**B**) Plots of internal energy (E), density (E_þ), temperature (T), pressure (P) and volume (v) of the entire system during 200 nsec simulation. The entire system of the simulation was investigated for the several parameters, as depicted in (**B**). During the 200 nsec simulation, the internal energy was found to be minimum and depicting a very stable conformation (**B**(E)), whereas the density was displayed to be lowered (**B**(E_þ)) and measured the compact conformation of the protein–ligand complex, while the temperature was found to be constant at 300K, as well as the pressure and volume of the entire system throughout the simulation time of 200 nsec (**B**(T,P,V)).

The docked complexes were studied for their conformation changes, stability and interaction profiles with respect to time (200 nsec). Figure 5A shows the detailed interaction of the key residues of PTP1B–silydianin along with the water molecule having >5.0% of the simulation time in the selected trajectory. After the 200 nsec simulation, the key residues Arg221, Thr263 and Asp265 were involved in making conventional hydrogen bonds with the silydianin ligand. The RMSD graph, as shown in Figure 5B, was observed to analyze the Cα atoms of the protein and was found to be stable and converged with an average deviation observed 0.5 Å from the beginning to the end of the 200 nsec simulation. However, the ligand silydianin RMSD displayed deviations at the early simulation time until 70 nsec, and thereafter became stable until 200 nsec (Figure 5B). Less RMS deviation of both protein and ligand were observed during the entire process of the 200 nsec simulation, indicating a stable and converged complex. The RMSF graph of the complex is depicted in Figure 5C. The RMSF of the Cα backbone of the protein displayed fluctuating residues at positions 40 and 240 at a maximum of 2.5–3 Å, and the rest of the positions did not fluctuate much, signifying that the binding of the ligand made a stable complex with the protein. Figure 5D shows the number of hydrogen bonds formed between the ligand and the protein during the 200 nsec of simulation time. Hydrogen bonding is the measure of the ligand stability in the complex formation. In this study, the average number of hydrogen bonds is displayed as formed by three numbers at the end of the simulation. The histogram in Figure 5E displayed the protein–ligand interactions/contacts are categorized into four types: H-bonds, hydrophobic, ionic and water bridges. Figure 6A,B show a detailed timeline representation of the protein–ligand interactions and contacts during the trajectory. The properties of ligands, such as the ligand RMSD, the radius of gyration, the intramolecular H-bonds, the molecular surface area, the solvent-accessible surface area and the polar surface area are shown in Figure 7. Figure 8 shows a comparison of the interaction of the docking results and the MD simulation result. Arg221 was observed to be the key residue participating in both environments.

### 2.5. Free Energy of Binding Analysis

**Figure 9:**
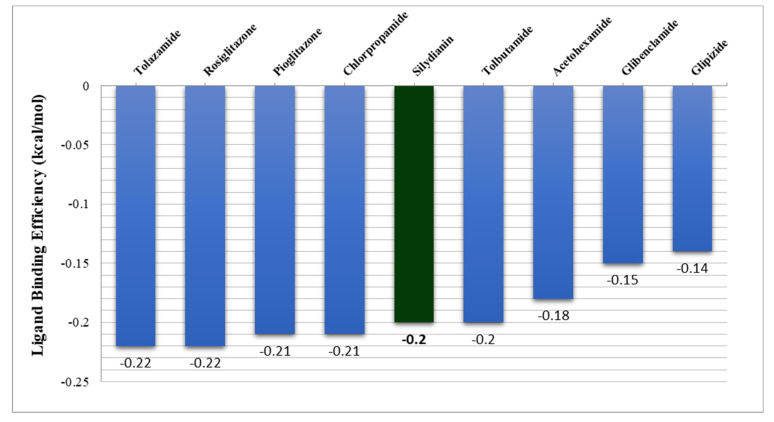
Comparison of the ligand-binding efficiency of natural polyphenol silydianin and synthetic drugs with PTP1B protein.

Table 3 shows the pKd, ΔG (kcal/mol) and ligand-binding efficiency (kcal/mol) of the natural polyphenols and the synthetic drugs. Silydianin was observed to have ΔG = −7.02 kcal/mol, pKd = 5.2 and a ligand-binding efficiency = −0.2 kcal/mol. The result was better as compared to the synthetic drugs tolbutamide, acetohexamide, glibenclamide and glipizide. However, the ligand-binding efficiency of silydianin was found to be similar to tolbutamide, i.e., −0.2 kcal/mol, while acetohexamide, glibenclamide and glipizide showed less negative ligand-binding efficiency, i.e., −0.18, −0.15 and −0.14 kcal/mol, respectively. A crucial factor used in the thermodynamic function of the binding free energy is the ligand torsion number as it relates to the flexibility of the ligand. It has an influence on the binding affinity prediction. The torsional energy is calculated by multiplying a weight factor (Wtor) with the torsion number of the ligand (Ntor). As the torsional energy (ΔG_torsion_) increases, it lowers the binding energy [38,39]. On comparing the torsional energy (ΔG_torsion_) of silydianin (+2.49 kcal/mol) with tolbutamide (+1.39 kcal/mol), but also the intermolecular energy as well as the van der Waals, the electrostatic and internal energies were much higher in the silydianin-bound protein complex, which minimized the high torsional energy penalty as compared to tolbutamide. Therefore, silydianin overcame the torsional penalty score and showed a better binding energy as compared to tolbutamide. Figure 9 depicts a comparison view of the ligand-binding efficiencies of the synthetic drugs and natural polyphenols with the PTP1B protein. The ligand-binding efficiency is calculated by using the structural information of both the protein and the respective ligand. Bioinformatic simulation-based approaches such as the methods used for free energy perturbation, ligand parametrization and force field selection have been focussed by the KDeep software [40]. 

## 3. Discussion

During previous years, many QSAR studies have been done to aid the analysis and design of novel PTB1B inhibitors [41]. In silico studies have shown flavonoids to possess PTP1B-inhibitory activity [42]. In this study, the QSAR models of the flavonoids with PTP1B were generated to analyze the correlations of the structural molecular descriptors with inhibitory activity. The PTP1B-inhibitory activity showed a maximum correlation of 93.25% with an increase in the number of aromatic-bond flavonoids. This was followed by the increase in the number of H-bond donors to 92.45%. The backbone structures of the polyphenols constitute aromatic entities which make them possess many beneficial biological implications [43,44,45]. Previous studies have revealed that the nature, position and number of substituents in the structure of the flavonoids play an important role in determining their ability to inhibit PTP1B [46,47]. Modifications in the B-ring in the flavonoids, and the presence of more nonpolar substituents, helps in increasing the ability of PTP1B inhibition [48,49]. In this study, the structural and activity relationship analysis using the QSAR models has shown that polyphenols (flavonoids) have an average of 14 aromatic bonds and that the increase in the aromatic bonds appears to play a role in the binding of the polyphenols with the PTP1B.

This study also aimed to identify polyphenols which could bind to the targeted site of the PTP1B protein and thereby hinder its mode of action. The grid box constructed contained the active site region of the catalytic domain of the PTP1B protein. Many polyphenols were able to bind to the catalytic active site with stable interactions and significant binding energies. Among all the polyphenols, silydianin was observed to be the top hit. It is a flavonolignan having a molecular weight of 482.4 g/mol and is commonly found in the plant parts of *Silybum marianum* (milk thistle) [33], which is a member of the Asteraceae family. It is an herb that has been used for medicinal purposes for more than 2000 years [50]. Recent work has shown silydianin to be a natural inhibitor of PTP1B [33]. In this paper, silydianin was found to be interacting with the active site of the PTP1B protein with a high negative docking score of −7.235 kcal/mol, an XPGScore of −7.268 kcal/mol and an MMGBSA of −63.42 kcal/mol in Schrödinger’s suite. The lead compound, silydianin, as reported by earlier studies conducted on in vivo rat models, human hepatocytes and pancreatic β-cells, has been studied to be nontoxic on hepatocytes and possess hepatoprotective effects. It does not lead to any cyto- and geno-toxicity in animals at 100 μM. It has been observed to be safe for use as a therapeutic drug for humans at a high dose of 700 mg thrice a day. Therefore, the compound can be effectively used as a therapeutic compound for ameliorating IR [51,52,53,54].

During the simulation it was observed that one positive-charged, one negative-charged, one polar amino acid and glycine formed bonds with the PTP1B. The interacting key residues are Gly183, Arg221, Thr263 and Asp265 (Figure 5A). The change in the average RMSD was observed to be 0.5 Å (between permissible limit, i.e., 1–3 Å). 

The protein–ligand RMSD graph (Figure 5B) suggests that the complex fluctuated between 1–2 Å within a time span of 0–70 nsecs. Comparing this phase to Figure 5D,E and Figure 6B suggests that Lys116, Trp179, Gly183, Val184, Cys215, Ser216, Ala217, Gly218, Gly220, Arg221, Gln262, Thr263 and Asp265 participated with the protein during this time interval and formed 3–6 H-bonds, as well as ionic bonds. The total contacts between PTP1B and silydianin varied from two to nine (Figure 6A).

Similarly, in the simultaneous analysis of the results obtained in Figure 5B,D,E with Figure 6B, we observed that from 70 to 140 nsecs, the complex was in close proximity. Residues of Glu76, Lys116, Asp181, Asp236, Lys237, Arg238 and Lys239 interacted in the initial duration, while Gln9, Ser13, Ser15, Ala17, Tyr20, Asp22 and Lys247 interacted in the later duration. Few H-bonds, water bridges and ionic bonds were observed during the duration. In the time interval 140–200 nsecs, the complex remained in close proximity and did not show many fluctuations. New bond formations with the residues Tyr20, Gln21, Arg24, Gln262, Thr263, Ala264 and Asp265 were observed. 

As seen in the Figure 5E histogram, Lys116, Gly183, Arg221, Gln262, Thr263 and Asp265 showed significant H-bonds and water bridges were formed by many amino acid residues, significantly by Glu6, Ser13, Gly14, Ala17, Asp22, Leu110, Cys215, Glu220, Ser243, Gly259 and Asp265. Significant ionic interactions were also observed with Cys215, Se216, Ala217, Gly218 and Ile219.

The entire ligand faced solvent exposure. The major interacting amino acids in the binding pocket, as per Figure 5A, were observed to be Gly183 (26%), Arg221 (23%), Thr263 (24%) and Asp265 (24% and 24%, respectively), which formed H-bonds with the ketone and hydroxyl groups of the ligand. Intramolecular interactions between the ketone and hydroxyl groups were also observed. The prevalence of H-bonds in the favored region play a significant role in any ligand-binding analysis because of their strong influence on drug specificity, metabolization and adsorption. It has therefore been used to analyze the potent inhibitors [11,12,55].

The comparison of the docking and MD simulation environments revealed the interaction of Arg221. Previous studies have shown Arg221 and Gln266 of the active site play a significant role in the conformational change of the WPD loop, and therefore have been targeted as a therapeutic target [56,57,58]. Lys116 is also one of the critical residues which provides PTP1B selectivity and plays a role in PTP1B inhibition [47,59]. The docking result of silydianin was also compared with some of the reported inhibitors (PDB ID: 5T19, 2QBP, 1T49 and 4I8N) and it was observed to be interacting with the residues of the active site. Arg221 and Gln266 were observed to be common interacting residues in silydianin and the reported inhibitors [60,61,62,63]. 

The molecular-free ligand-binding analysis of silydianin showed a pKd value of 5.2, a ΔG value of −7.02 kcal/mol and a ligand-binding efficiency of −0.2 kcal/mol, and showed a better binding energy than many synthetic drugs included in the study. Rosiglitazone (a synthetic drug), with a molecular weight of 357.428 g/mol, showcased a reduced docking score of −5.432 kcal/mol, a XPGScore of −5.999 kcal/mol and an MMGBSA of −38.73 kcal/mol as compared to top hit selected polyphenols. It formed stable H-bonds with the amino and ketone groups of amino acids Lys120, Trp179, Gly183, Arg221 and Gln266 and Pi-alkyl bonds with Lys116.

**Figure 10:**
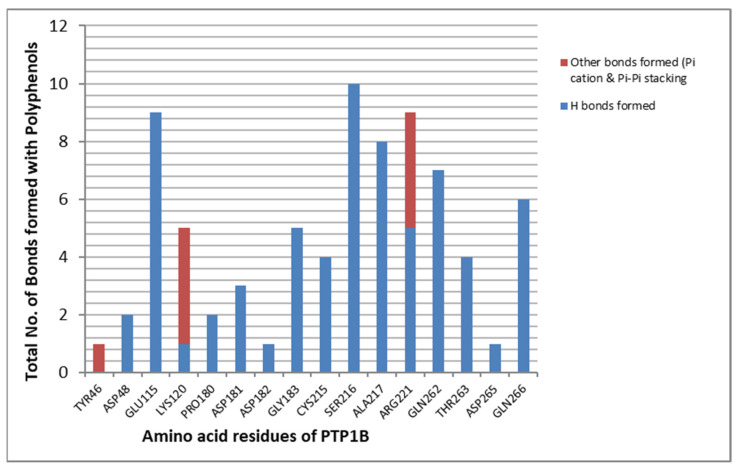
Graph showing amino acid residues of PTP1B and maximum number of bonds they formed with the respective amino acids.

Most of the polyphenols included in this study were observed to have better ligand-binding energies as compared to many of the synthetic drug candidates. The number of bonds formed with targeted amino acid residues in the catalytic active cavity and WPD loop, such as Tyr46, Lys120, Trp179, Asp181, Gly183, Cys215, Ser216, Ala217, Arg221, Gln262, Thr263 and Gln266, are shown in Figure 10. As studied earlier, these amino acid residues play a crucial role in the functioning of the protein. The presence of many polar amino acid residues and a shallow catalytic pocket selectively binds with small molecules with polar groups and makes the discovery of inhibitors a challenging task [64,65]. Inhibitors targeting the active site have been observed to inhibit the protein [14]. Hence, the stable binding of our polyphenols with the active site could hinder the functioning of the protein.

The polyphenols could be explored more for their activity and could act as a natural drug candidate in the replacement of synthetic drugs. These polyphenols bind to the active site of the protein. The binding may hinder its phosphatase action in the cell and lead to the normal regulation of the insulin signaling pathway, thereby proving to be a therapeutic drug candidate for treating T2DM.

## 4. Materials and Methods

### 4.1. QSAR Model Analysis

Experimental IC_50_ values of a list of 46 antidiabetic flavonoids with the PTP1B protein were retrieved from literature reviews. Molecular descriptors of each flavonoid were derived as shown in Table 4. A series of rigorous sorting and division was done using each of the descriptor parameters to prepare an unbiased training set and test set in four ratios (50:50, 70:30, 75:25 and 25:75), as shown in Table 5. The activities and descriptor data were loaded in the respective fields of the training set of the EasyQSAR software version 1.0.0.0 for multiple linear regression analysis [66]. From the regression, the QSAR equation was generated and the activities for each molecule were predicted. To keep the mean deviation of the data small, the LogIC_50_ value was used as the criteria to predict the biological activity. The model generated was then tested by substituting the test set molecular descriptor data in the respective fields. The LogIC_50_ values were predicted for the test set data.

### 4.2. Database Generation and Ligand Preparation

The 3-D conformers/chemical files of 173 antidiabetic phytochemicals/ligands in .sdf format were retrieved using the National Center for Biotechnology Information–PubChem and a database of all the ligands was generated. Human PTP1B with a PDB Id: 3A5J model having 327 residues was retrieved in .pdb format from the RCSB Protein Data Bank. The chemical files of all the shortlisted polyphenols/ligands were converted from .sdf to .pdbqt format using the Open Babel tool version 2.4.1 [80].

### 4.3. Evaluation of Drug-Likeness

To prioritize the drug-like phytochemicals, it is necessary to screen molecules based on certain filters. Drug-likeness is a set of rules/guidelines (such as Lipinski’s rule) for the structural properties of compounds/ligands which are used for the fast calculation of the drug-like properties of a molecule. DruLiTo software was used to apply Lipinski’s rule of five to all the phytochemicals. The 3-D conformers of the ligands in .sdf format were uploaded in the software and subjected to Lipinski’s rule [81]. The resultant ligands qualifying through the software were shortlisted and carried for further analysis.

### 4.4. Preparation for Target Protein

#### 4.4.1. Preparation of Receptor PTP1B Protein

The 3-D structure was visualized in the Biovia Discovery Studio Visualizer 2019 client. Water molecules and heteroatom/ions were removed. Energy minimization with 50:50 (steepest descent/conjugate gradient) was carried out using the Swiss PDB Viewer (SPDV) version 4.10 and Chimera 1.6.2. The protein receptor was visually analyzed to check for any deformation. Ramachandran plot analysis of the protein revealed that the amino acids occupied the allowed regions. The resultant minimized protein was converted from .pdb to .pdbqt format using the Open Babel tool version 2.4.1.

#### 4.4.2. Identification of Active Site and Preparation of Grid Box

A literature review was done to identify the active sites of the catalytic domain of the protein. The active site included Cys215; catalytic loop His214, Ser216, Ala217, Gly218, Ile219, Gly220 and Arg221; catalytic WPD loop Trp179, Pro180 and Asp181 [11]. The other key residues of the catalytic cavity included Tyr46, Asp48, Phe182, Gly183, Ile219, Gln220, Gln262 and Gln266 [57]. The grid box was prepared along these selected sites.

### 4.5. Docking Using Schrödinger Suite

The docking of each DruLiTo-qualified ligand and the PTP1B protein active site was carried out using Schrödinger suite version 3.4. All the shortlisted ligands were subjected to the Glide module of Schrödinger’s suite based on a three-tiered docking strategy. The docking was carried out using two separate strategies/techniques. In the first technique, the ligands–protein interaction was virtually screened through high-throughput virtual screening (HTVS). A total of 50% of the above-qualified ligands underwent standard precision (SP) docking while 50% of the SP-docked ligands were subjected to extra precision (XP) docking. The second technique consisted of the docking and screening of all the ligands directly to the XP docking. All other software parameters were maintained as default. The binding energies were calculated of all the interactions that were obtained. The results obtained through both methods were compiled and the polyphenols were carried for further analysis.

### 4.6. Molecular Dynamics and Simulation of Silydianin and PTP1B Complex

The best docked pose of the PTP1B protein and ligand (silydianin) complex was subjected to MD simulation with the default setting for evaluating the complex stability by 200 nsec without any restraints. MD simulation was performed for 200 nsec using the Desmond 2020.1 from Schrödinger, LLC. The OPLS-2005 force field [82,83,84] and explicit solvent model with the SPC water molecules were used in this system [85]. The protein chain ‘A’ with 282 total residues possessed 4595 atoms, 2307 heavy atoms and +2 charges. Here, chain A is mentioned as the X-ray structure of the protein in the PDB databank and contains the information of that particular chain only. The ligand with the atomic mass of 500.5 au had 75 atoms, 35 heavy atoms and 0 charge. The protein–ligand complex having overall 16,747 atoms was subjected to a periodic boundary condition within a grid box with the desired dimensions of 10 Å × 10 Å × 10 Å. A total of 4018 water molecules were added for system minimization. An amount of 11 sodium (Na^+1^) and 13 chlorine (Cl^−1^) was added to neutralize the overall charge of the complex and 0.15 M NaCl solutions were added to the system to simulate the physiological environment. The NPT ensemble was set up using the Nose–Hoover chain coupling scheme [86] with the temperature of 27 °C, the relaxation time of 1.0 psec and the pressure of 1 bar maintained in all the simulations. A time step of 2 fsec was used. The Martyna–Tuckerman–Klein chain coupling scheme [87] barostat method was used for pressure control with a relaxation time of 2 psec. The particle mesh Ewald method [88] was used for calculating the long-range electrostatic interactions, and the radius for the Coulomb interactions were fixed at 9 Å. The RESPA integrator was used to calculate the nonbonded forces. The root mean square deviation (RMSD) was employed to monitor the stability of the MD simulations. The trajectories of the simulation and interactions were analyzed using Desmond Maestro version 12.5.

## 5. Conclusions

This study was based on finding the few polyphenols which could interact with the active site of the PTP1B protein and hinder its phosphatase action. Silydianin was found to have a highly negative binding energy that spontaneously binds to the active site of the protein by forming stable bonds. Occupying the active site of the phosphatase protein would reduce the ability of the protein to bind to the insulin receptors and dephosphorylate the Tyr residues [13,58] As a result, the normal insulin signaling pathway would remain active and the downstream signaling process would lead to the uptake of glucose by the cells. It showed better results as compared to the synthetic drug compounds used in the study. A correlation between the number of aromatic bonds in the flavonoids and the PTP1B inhibition properties was seen using the model generated through QSAR. Further detailed analysis of the molecular mechanism of the action of the polyphenol and its dosage will be carried out to observe its effect on insulin resistance.

## Figures and Tables

**Table 1:** Correlation analysis table of QSAR model of increase in number of aromatic bonds and PTP1B inhibition.

Training Set (Increase in Number of Aromatic Bonds)			
Ratio	R^2^	Adjusted R^2^	R^2^–Adjusted R^2^
50:50	19.89%	−17.50%	37.39
70:30	32.67%	13.03%	19.64
75:25	36.11%	18.91%	17.20
25:75	93.25%	81.43%	11.82
**Test Set (Increase in Number of Aromatic Bonds)**			
Ratio	R^2^	Adjusted R^2^	R^2^–Adjusted R^2^
50:50	19.49%	−15.51%	35.00%
70:30	30.74%	13.21%	17.53%
75:25	33.04%	19.44%	13.60%
25:75	91.12%	81.40%	9.72%

**Table 2:** Docking results using Schrödinger’s suite. (**A**) Docking based virtual screening (**B**) Extra precision docking.

(A) Virtual Screening Workflow Docking						
S. No.	Ligands	Structure	Present in	Docking Score (kcal/mol)	XPGScore (kcal/mol)	MMSGBA (kcal/mol)
1	Silydianin	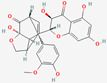	*Silybum marianum*	−7.23	−7.26	−63.42
2	Morin	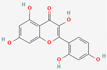	*Morus alba*	−6.73	−6.98	−47.10
3	Cianidanol	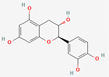	*Punica granatum*	−6.21	−6.21	−46.93
4	Rosmarinic acid	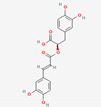	*Lamiaceae*	−6.14	−6.14	−46.72
5	Leucopelargonidin	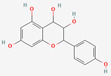	*Ficus bengalensis*	−6.03	−6.03	−48.25
6	(-)-Epicatechin	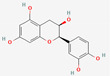	*Pterocarpus marsupium*	−6.00	−6.00	−46.57
7	Apigenin	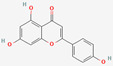	*Matricaria recutita*	−5.84	−5.88	−38.81
8	Wogonin	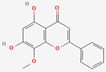	*Scutellaria baicalensis*	−5.78	−5.82	−40.49
9	Malic acid	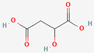	*Syzygium cumini*	−5.78	−5.78	−28.17
10	Luteolin	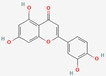	*Capsicum*	−5.51	−5.55	−38.73
11	Rosiglitazone (drug)	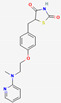		−5.43	−5.99	−47.24
12	Cajanin	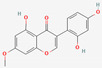	*Cajanus cajan*	−5.34	−5.34	−56.52
**(B) Extra Precision Docking**						
**S. No.**	**Ligands**	**Structure**	**Present in**	**XP Score (kcal/mol)**		
1	Rosmarinic acid	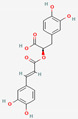	*Lamiaceae*	−8.21		
2	Ellagic acid	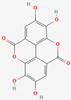	*Syzygium cumini*	−7.36		
3	Morin	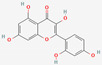	*Morus alba*	−7.23		
4	Silydianin	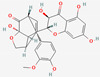	*Silybum marianum*	−7.17		
5	Salacinol	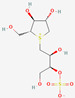	*Salacia reticulate*	−6.96		
6	Curcumin	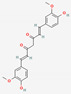	*Curcuma longa*	−6.84		
7	Baicalein	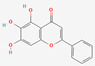	*Scutellaria baicalensis*	−6.67		
8	Miglitol (drug)	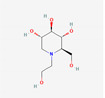		−6.67		

**Table 3:** KDeep analysis of shortlisted natural polyphenols and synthetic drugs.

S. No.	Ligands	Mol. Weight g/mol	pKd (Std.)	ΔG kcal/mol (Std.)	Ligand-Binding Efficiency kcal/mol (Std.)
1	Catechol	110.04	3.62 (0.17)	−4.89 (−0.24)	−0.61 (−0.03)
2	Metformin	129.10	3.56 (0.49)	−4.81 (−0.66)	−0.53 (0.07)
3	(-)-Epicatechin	290.08	6.01 (0.40)	−8.12 (−0.54)	−0.39 (−0.03)
4	Cianidanol	290.08	5.75 (0.20)	−7.76 (−0.27)	−0.37(−0.01)
5	Salacinol	334.04	5.28 (0.71)	−7.13 (−0.95)	−0.36 (−0.05)
6	Luteolin	286.05	5.38 (0.40)	−7.26 (−0.54)	−0.35(−0.03)
7	Topiramate	339.10	5.52 (0.56)	−7.46 (−0.76)	−0.34 (−0.03)
8	Baicalein	270.05	5.00 (0.45)	−6.75 (−0.06)	−0.34 (−0.03)
9	Morin	302.04	5.46 (0.36)	−7.37 (−0.48)	−0.34 (−0.02)
10	Ellagic acid	302.01	5.24 (0.78)	−7.08 (−1.06)	−0.32 (−0.05)
11	Cajanin	300.06	5.07 (0.28)	−6.85 (−0.37)	−0.31 (−0.02)
12	Apigenin	270.05	4.54 (0.47)	−6.13 (−0.64)	−0.31 (−0.03)
13	Leucopelargonidin	290.08	4.85 (0.43)	−6.55 (−0.58)	−0.31 (−0.03)
14	Wogonin	284.07	4.62 (0.46)	−6.24 (−0.62)	−0.30 (−0.03)
15	Malic acid	134.02	1.95 (0.39)	−2.64 (−0.53)	−0.29 (−0.06)
16	Rosmarinic acid	360.08	5.41 (0.66)	−7.30 (−0.90)	−0.28 (−0.03)
17	Curcumin	368.13	5.43 (0.76)	−7.33 (−1.02)	−0.27 (−0.04)
18	Brevifolin carboxylic acid	292.02	3.42 (0.44)	−4.61 (−0.60)	−0.22 (−0.03)
19	Tolazamide	311.13	3.40 (0.49)	−4.59 (−0.66)	−0.22 (−0.03)
20	Rosiglitazone	357.11	4.16 (0.57)	−5.62 (−0.77)	−0.22 (−0.03)
21	Pioglitazone	356.12	3.87 (0.48)	−5.23 (−0.64)	−0.21 (−0.03)
22	Chlorpropamide	276.03	2.65 (0.17)	−3.57 (−0.23)	−0.21 (−0.01)
23	Silydianin	482.12	5.20 (0.35)	−7.02 (−0.47)	−0.20 (−0.01)
24	Tolbutamide	270.10	2.70 (0.24)	−3.64 (−0.32)	−0.20 (−0.02)
25	Acetohexamide	324.11	2.88 (0.42)	−3.89 (−0.57)	−0.18 (−0.03)
26	Glibenclamide	493.14	3.61 (0.43)	−4.87 (−0.58)	−0.15 (−0.02)
27	Glipizide	445.18	3.31 (0.38)	−4.47 (−0.51)	−0.14 (−0.02)

**Table 4:** Antidiabetic flavonoids with molecular descriptors.

S. No.	Compound Name	IC_50_ (μM)	LogIC_50_	Mol. Wt.	Hydrogen Bond Acceptor/HBA (Naccr)	Hydrogen Bond Donor/HBD (Ndonr)	No. of Rotatable Bonds (Nrot)	No. of Aromatic Bonds (Naro)	Topological Polar Surface Area (TPSA)	LogP	References
1	Viscosol	13.5	1.13	388.246	7	2	6	17	98.36	4.406	[67]
2	Penduletin	57.9 ± 0.6	1.762	328.191	7	2	4	17	98.36	2.897	[67]
3	5,6-Dihydroxy-3,4′,7-trimethoxyflavone	32.2 ± 0.8	1.507	328.191	7	2	4	17	98.36	2.897	[67]
4	Kaempferol 3-O-rutinoside	20.5 ± 0.8	1.311	564.282	15	9	6	17	249.2	−1.393	[67]
5	Isorhamnetin3-O-robinobioside	42.9 ± 0.4	1.632	592.292	16	9	7	17	258.43	−1.384	[67]
6	Erybraedin A	2.4 ± 0.7	0.38	364.271	4	2	4	12	58.92	5.725	[27]
7	Luteolin	6.70 ± 0.03	0.826	276.159	6	4	1	17	111.13	2.282	[68]
8	2′-Methoxykurarinone	5.26 ± 0.24	0.72	420.291	6	2	8	12	85.22	5.913	[68]
9	Mulberrofuran D	4.3 ± 0.5	0.633	412.315	4	3	8	16	73.83	7.961	[69]
10	Mulberrofuran W	2.7 ± 0.3	0.431	412.315	4	3	9	16	73.83	8.178	[69]
11	Catechin	2.245	0.351	276.159	6	5	1	12	110.38	1.546	[70]
12	Epicatechin	0.832	−0.079	276.159	6	5	1	12	110.38	1.546	[70]
13	Trans-resveratrol	16.1 ± 1.1	1.206	216.151	3	3	2	12	60.69	2.974	[71]
14	Apigenin	24.76	1.393	260.16	5	3	1	17	90.9	2.577	[71]
15	Isovitexin	17.76	1.249	412.221	10	7	3	17	181.05	0.092	[72]
16	Vitexin	7.62	0.881	412.221	10	7	3	17	181.05	0.092	[72]
17	Isoorientin	24.54	1.389	428.22	11	8	3	17	201.28	−0.203	[72]
18	Orientin	57.11	1.756	428.22	11	8	3	17	201.28	−0.203	[72]
19	abyssinin II/5′-Prenylhomoeriodictyol	40.5 ± 1.9	1.607	348.225	6	3	4	12	96.22	4.027	[73]
20	Parvisoflavone B	42.6 ± 2.4	1.629	336.214	6	3	1	17	100.13	3.761	[73]
21	Neorautenol	7.6 ± 0.9	0.88	304.216	4	1	0	12	47.92	4.186	[69]
22	Erybreadin D	4.2 ± 0.2	0.623	364.271	4	1	2	12	47.92	5.695	[27]
23	Erybreadin B	7.8 ± 0.5	0.892	364.271	4	1	2	12	47.92	5.695	[27]
24	Folitenol	6.4 ± 0.6	0.806	364.271	4	1	2	12	47.92	5.695	[74]
25	Erysubin E	8.8 ± 0.5	0.944	380.27	5	2	2	12	68.15	4.799	[74]
26	Erybreadin C	7.3 ± 0.1	0.863	364.271	4	2	4	12	58.92	5.725	[74]
27	Licoagrone	6.0	0.778	700.485	10	5	10	24	170.82	8.71	[27]
28	Erythraddison III	4.6 ± 0.1	0.662	332.226	5	2	4	12	75.99	3.974	[27]
29	Erysubin F	7.8 ± 0.5	0.892	364.271	4	2	5	17	70.67	5.889	[27]
30	2′-Methoxykurarinone	5.26 ± 0.24	0.72	420.291	6	2	8	12	85.22	5.913	[27]
31	Mimulone/ Bonannione A	1.9 ± 0.1	0.278	380.27	5	3	6	12	86.99	5.745	[75]
32	3′-O-Methyldiplacone	3.9 ± 0.3	0.591	408.28	6	3	7	12	96.22	5.754	[75]
33	6-geranyl-3′,5,5′,7-Tetrahydroxy-4′-methoxyflavanone	5.9 ± 0.4	0.77	424.279	7	4	7	12	116.45	5.459	[75]
34	4′-O-methyldiplacone	7.8 ± 0.6	0.892	408.28	6	3	7	12	96.22	5.754	[75]
35	3′-O-methyldiplacol	4.9 ± 0.5	0.69	424.279	7	4	7	12	116.45	4.725	[75]
36	4′-O-methyldiplacol	8.2 ± 0.6	0.913	424.279	7	4	7	12	116.45	4.725	[75]
37	6-geranyl-3,3′,5,5′,7-Pentahydroxy-4′-methoxyflavane	6.6 ± 0.5	0.819	424.279	7	5	7	12	119.61	4.79	[75]
38	Laxichalcone	20.7 ± 5.3	1.315	380.27	5	2	3	12	75.99	5.362	[76]
39	Macarangin	22.7 ± 4.6	1.356	396.269	6	4	6	17	111.13	5.518	[76]
40	Bonanniol A	15.2 ± 2.8	1.181	396.269	6	4	6	12	107.22	4.716	[76]
41	7,4′-Dimethylkaempferol/3,5-Dihydroxy-7-methoxy-2-(4-methoxyphenyl)-4H-chromen-4-one	16.92 ± 2.12	1.228	300.181	6	2	3	17	89.13	2.888	[76]
42	2S-5,6,7,3′,4′-Pentamethoxyflavanone	6.88 ± 0.76	0.837	352.213	7	0	6	12	72.45	3.436	[77]
43	3′-Hydroxy-3,5,7,4′-tetramethoxyflavone	22.25 ± 1.70	1.347	340.202	7	1	5	17	87.36	3.2	[77]
44	3,5-Dihydroxy-7,3′,4′-trimethoxyflavone	52.64 ± 4.12	1.721	328.191	7	2	4	17	98.36	2.897	[77]
45	Lutein	13.691	1.136	512.438	2	2	10	1	40.46	10.403	[78]
46	Silydianin	17.38	1.24	460.265	10	5	3	12	162.98	0.99	[79]

**Table 5:** Sorting and division method using each descriptor parameter to prepare an unbiased training set and test set in four ratios (50:50, 70:30, 75:25 and 25:75).

	Total Compounds = 46		
S. No.	Sorting and Division Methods	Training Set Compounds	Test Set Compounds
1	Random Sorting:- I (50:50)	1–23	24–46
	II (70:30)	1–32	33–46
	III(75:25)	1–34	35–46
	IV(25:75)	1–12	13–46
2	Increasing Mol. Weight:- I(50:50)	13, 14, 7, 11, 12, 41, 21, 2, 3, 44, 28, 20, 43, 19, 42, 6, 22, 23, 24, 26, 29, 25, 31	38, 1, 39, 40, 32, 34, 15, 16, 9, 10, 8, 30, 33, 35, 36, 37, 17, 18, 46, 45, 4, 5, 27
	II (70:30)	13, 14, 7, 11, 12, 41, 21, 2, 3, 44, 28, 20, 43, 19, 42, 6, 22, 23, 24, 26, 29, 25, 31, 38, 1, 39, 40, 32, 34, 15, 16, 9	10, 8, 30, 33, 35, 36, 37, 17, 18, 46, 45, 4, 5, 27
	III(75:25)	13, 14, 7, 11, 12, 41, 21, 2, 3, 44, 28, 20, 43, 19, 42, 6, 22, 23, 24, 26, 29, 25, 31, 38, 1, 39, 40, 32, 34, 15, 16, 9, 10, 8	30, 33, 35, 36, 37, 17, 18, 46, 45, 4, 5, 27
	IV(25:75)	13, 14, 7, 11, 12, 41, 21, 2, 3, 44, 28, 20	43, 19, 42, 6, 22, 23, 24, 26, 29, 25, 31, 38, 1, 39, 40, 32, 34, 15, 16, 9, 10, 8, 30, 33, 35, 36, 37, 17, 18, 46, 45, 4, 5, 27
3	Increasing H-bond Acceptor (HBA) (naccr):- I(50:50)	45, 13, 6, 9, 10, 21, 22, 23, 24, 26, 29, 14, 25, 28, 31, 38, 7, 8, 11,12, 19, 20, 30	32, 34, 39, 40, 41, 1, 2, 3, 33, 35, 36, 37, 42, 43, 44, 15, 16, 27, 46, 17, 18, 4, 5
	II (70:30)	45, 13, 6, 9, 10, 21, 22, 23, 24, 26, 29, 14, 25, 28, 31, 38, 7, 8, 11,12, 19, 20, 30, 32, 34, 39, 40, 41, 1, 2, 3, 33	35, 36, 37, 42, 43, 44, 15, 16, 27, 46, 17, 18, 4, 5
	III (75:25)	45, 13, 6, 9, 10, 21, 22, 23, 24, 26, 29, 14, 25, 28, 31, 38, 7, 8, 11,12, 19, 20, 30, 32, 34, 39, 40, 41, 1, 2, 3, 33, 35, 36	37, 42, 43, 44, 15, 16, 27, 46, 17, 18, 4, 5
	IV (25:75)	45, 13, 6, 9, 10, 21, 22, 23, 24, 26, 29, 14	25, 28, 31, 38, 7, 8, 11,12, 19, 20, 30, 32, 34, 39, 40, 41, 1, 2, 3, 33, 35, 36, 37, 42, 43, 44, 15, 16, 27, 46, 17, 18, 4, 5
4	Increasing H-bond Donor (HBD) (ndonr):- I(50:50)	42, 21, 22, 23, 24, 43, 1, 2, 3, 6, 8, 25, 26, 28, 29, 30, 38, 41, 44, 45, 9, 10, 13	14, 19, 20, 31, 32, 34, 7, 33, 35, 36, 39, 40, 11, 12, 27, 37, 46, 15, 16, 17, 18, 4, 5
	II (70:30)	42, 21, 22, 23, 24, 43, 1, 2, 3, 6, 8, 25, 26, 28, 29, 30, 38, 41, 44, 45, 9, 10, 13, 14, 19, 20, 31, 32, 34, 7, 33, 35	36, 39, 40, 11, 12, 27, 37, 46, 15, 16, 17, 18, 4, 5
	III (75:25)	42, 21, 22, 23, 24, 43, 1, 2, 3, 6, 8, 25, 26, 28, 29, 30, 38, 41, 44, 45, 9, 10, 13, 14, 19, 20, 31, 32, 34, 7, 33, 35, 36, 39	40, 11, 12, 27, 37, 46, 15, 16, 17, 18, 4, 5
	IV (25:75)	42, 21, 22, 23, 24, 43, 1, 2, 3, 6, 8, 25	26, 28, 29, 30, 38, 41, 44, 45, 9, 10, 13, 14, 19, 20, 31, 32, 34, 7, 33, 35, 36, 39, 40, 11, 12, 27, 37, 46, 15, 16, 17, 18, 4, 5
5	Increasing No. of Rotatable Bonds (nrot):- I(50:50)	21, 7, 11, 12, 14, 20, 13, 22, 23, 24, 25, 15, 16, 17, 18, 38, 41, 46, 2, 3, 6, 19, 26	28, 44, 29, 43, 1, 4, 31, 39, 40, 42, 5, 32, 33, 34, 35, 36, 37, 8, 9, 30, 10, 27, 45
	II (70:30)	21, 7, 11, 12, 14, 20, 13, 22, 23, 24, 25, 15, 16, 17, 18, 38, 41, 46, 2, 3, 6, 19, 26, 28, 44, 29, 43, 1, 4, 31, 39, 40	42, 5, 32, 33, 34, 35, 36, 37, 46, 8, 9, 30, 10, 27, 45
	III (75:25)	21, 7, 11, 12, 14, 20, 13, 22, 23, 24, 25, 15, 16, 17, 18, 38, 41, 46, 2, 3, 6, 19, 26, 28, 44, 29, 43, 1, 4, 31, 39, 40, 42, 5	32, 33, 34, 35, 36, 37, 46, 8, 9, 30, 10, 27, 45
	IV (25:75)	21, 7, 11, 12, 14, 20, 13, 22, 23, 24, 25, 15,	16, 17, 18, 38, 41, 46, 2, 3, 6, 19, 26, 28, 44, 29, 43, 1, 4, 31, 39, 40, 42, 5, 32, 33, 34, 35, 36, 37, 46, 8, 9, 30, 10, 27, 45
6	Increasing No. of Aromatic Bonds (naro):- I(50:50)	45, 6, 8, 11, 12, 13, 19, 21, 22, 23, 24, 25, 26, 28, 30, 31, 32, 33, 34, 35, 36, 37, 38	40, 42, 46, 9, 10, 1, 2, 3, 4, 5, 7, 14, 15, 16, 17, 18, 20, 29, 39, 41, 43, 44, 27
	II (70:30)	45, 6, 8, 11, 12, 13, 19, 21, 22, 23, 24, 25, 26, 28, 30, 31, 32, 33, 34, 35, 36, 37, 38, 40, 42, 46, 9, 10, 1, 2, 3, 4	5, 7, 14, 15, 16, 17, 18, 20, 29, 39, 41, 43, 44, 27
	III (75:25)	45, 6, 8, 11, 12, 13, 19, 21, 22, 23, 24, 25, 26, 28, 30, 31, 32, 33, 34, 35, 36, 37, 38, 40, 42, 46, 9, 10, 1, 2, 3, 4, 5, 7	14, 15, 16, 17, 18, 20, 29, 39, 41, 43, 44, 27
	IV (25:75)	45, 6, 8, 11, 12, 13, 19, 21, 22, 23, 24, 25	26, 28, 30, 31, 32, 33, 34, 35, 36, 37, 38, 40, 42, 46, 9, 10, 1, 2, 3, 4, 5, 7, 14, 15, 16, 17, 18, 20, 29, 39, 41, 43, 44, 27
7	Increasing Topological Polar Surface Area (TPSA):- I(50:50)	45, 21, 22, 23, 24, 6, 26, 13, 25, 29, 42, 9, 10, 28, 38, 8, 30, 31, 43, 41, 14, 19, 32	34, 1, 2, 3, 44, 20, 40, 11, 12, 7, 39, 33, 35, 36, 37, 46, 27, 15, 16, 17, 18, 4, 5
	II (70:30)	45, 21, 22, 23, 24, 6, 26, 13, 25, 29, 42, 9, 10, 28, 38, 8, 30, 31, 43, 41, 14, 19, 32, 34, 1, 2, 3, 44, 20, 40, 11, 12	7, 39, 33, 35, 36, 37, 46, 27, 15, 16, 17, 18, 4, 5
	III (75:25)	45, 21, 22, 23, 24, 6, 26, 13, 25, 29, 42, 9, 10, 28, 38, 8, 30, 31, 43, 41, 14, 19, 32, 34, 1, 2, 3, 44, 20, 40, 11, 12, 7, 39	33, 35, 36, 37, 46, 27, 15, 16, 17, 18, 4, 5
	IV (25:75)	45, 21, 22, 23, 24, 6, 26, 13, 25, 29, 42, 9	10, 28, 38, 8, 30, 31, 43, 41, 14, 19, 32, 34, 1, 2, 3, 44, 20, 40, 11, 12, 7, 39, 33, 35, 36, 37, 46, 27, 15, 16, 17, 18, 4, 5
8	Increasing LogP:- I(50:50)	4, 5, 17, 18, 15, 16, 46, 11, 12, 7, 14, 41, 2, 3, 44, 13, 43, 42, 20, 28, 19, 21, 1	40, 35, 36, 37, 25, 38, 33, 39, 22, 23, 24, 6, 26, 31, 32, 34, 39, 8, 30, 9, 10, 27, 45
	II (70:30)	4, 5, 17, 18, 15, 16, 46, 11, 12, 7, 14, 41, 2, 3, 44, 13, 43, 42, 20, 28, 19, 21, 1, 40, 35, 36, 37, 25, 38, 33, 39, 22	23, 24, 6, 26, 31, 32, 34, 39, 8, 30, 9, 10, 27, 45
	III (75:25)	4, 5, 17, 18, 15, 16, 46, 11, 12, 7, 14, 41, 2, 3, 44, 13, 43, 42, 20, 28, 19, 21, 1, 40, 35, 36, 37, 25, 38, 33, 39, 22, 23, 24	6, 26, 31, 32, 34, 39, 8, 30, 9, 10, 27, 45
	IV (25:75)	4, 5, 17, 18, 15, 16, 46, 11, 12, 7, 14, 41	2, 3, 44, 13, 43, 42, 20, 28, 19, 21, 1, 40, 35, 36, 37, 25, 38, 33, 39, 22, 23, 24, 6, 26, 31, 32, 34, 39, 8, 30, 9, 10, 27, 45

## Data Availability

Not applicable.

## Abbreviations

GLUT-4	Glucose transport channel 4
HBA	Hydrogen bond acceptor
HBD	Hydrogen bond donor
IR	Insulin receptors
IRS	Insulin receptor substrates
PDB	Protein data bank
PTP1B	Protein tyrosine phosphatase 1B
RMSD	Root mean square deviation
T2DM	Type 2 diabetes mellitus
